# A Critical Review of the Prognostic and Predictive Implications of *KRAS* and *STK11* Mutations and Co-Mutations in Metastatic Non-Small Lung Cancer

**DOI:** 10.3390/jpm13061010

**Published:** 2023-06-18

**Authors:** Peter Manolakos, Linda D. Ward

**Affiliations:** Healthcare Genetics and Genomics PhD Program, Clemson University, Clemson, SC 29634, USA; ldward@clemson.edu

**Keywords:** *KRAS*, mutations, *STK11*, co-mutation, *KRAS/STK11*, *KL*, prognostic, predictive, NSCLC, non-small cell lung cancer

## Abstract

The Kirsten rat sarcoma viral oncogene homolog (*KRAS*) and serine/threonine kinase 11 (*STK11*) co-mutations are associated with the diverse phenotypic and heterogeneous oncogenic subtypes in non-small cell lung cancer (NSCLC). Due to extensive mixed evidence, there needs to be a review of the recent *KRAS* and *STK11* mutation literature to better understand the potential clinical applications of these genomic biomarkers in the current treatment landscape. This critical review highlights the clinical studies that have elucidated the potential prognostic and predictive implications of *KRAS* mutations, *STK11* mutations, or *KRAS/STK11* co-mutations when treating metastatic NSCLC across various types of treatments (e.g., immune checkpoint inhibitors [ICIs]). Overall, *KRAS* mutations are associated with poor prognoses and have been determined to be a valid but weak prognostic biomarker among patients diagnosed with NSCLC. *KRAS* mutations in NSCLC have shown mixed results as a predictive clinical biomarker for immune checkpoint inhibitor treatment. Overall, the studies in this review demonstrate that *STK11* mutations are prognostic and show mixed results as predictive biomarkers for ICI therapy. However, *KRAS/STK11* co-mutations may predict primary resistance to ICI. Prospective *KRAS/STK11*-biomarker-driven randomized trials are needed to assess the predictive effect of various treatments on the outcomes for patients with metastatic NSCLC, as the majority of the published *KRAS* analyses are retrospective and hypothesis-generating in nature.

## 1. Introduction

Lung cancer is the most common cause of cancer mortality globally, with 1.8 million deaths annually, and has one of the lowest five-year survival rates at approximately 23% [1,2]. Non-small lung cancer (NSCLC) predominates among lung cancer sub-types, representing 85% of cases, with over 40% of NSCLC patients having adenocarcinoma [3,4]. In the twenty-first century, identifying the molecular subsets of lung adenocarcinoma characterized by specific oncogenic drivers (e.g., epidermal growth factor receptor [*EGFR*]) has led to remarkable improvements in the treatments and outcomes for various lung cancers with tyrosine kinase inhibitors (TKI) [5]. However, one of the most prominent oncogenic drivers of lung cancer identified to date has eluded the discovery of effective therapies. *KRAS* (Kirsten rat sarcoma viral oncogene homolog) oncogene mutations are the most frequently reported gain-of-function driver mutations in NSCLC. They are found in 30–35% of NSCLC adenocarcinomas, with the *KRAS* G12C variant being the most common [6]. In a heterogeneous meta-analysis of 23 NSCLC chemotherapy-based studies, 2631 patients with *KRAS* mutations and an adenocarcinoma subtype had poor prognoses, with a statistically worse overall survival than patients without *KRAS* mutations [7]. Instead of solely focusing on chemotherapy, other compounds with various mechanisms of action were evaluated to indirectly target *KRAS* via downstream pathways [8]. These treatments did not improve survival in prospective studies, suggesting that *KRAS*-mutated proteins may need to be targeted directly [8].

Since 2016, the National Comprehensive Cancer Network (NCCN) has recommended next-generation sequencing (NGS) for metastatic non-squamous NSCLC [9]. Tumor sequencing has led to improved detection of molecular aberrations and the delivery of precision medicine. One crucial real-world outcome of increased NGS was learning that *KRAS* G12C commonly co-occurs with other pathogenic variants upon its diagnosis, which differs from the other established NSCLC driver mutations (e.g., *EGFR*) [10]. In NSCLC, the co-occurrence of *KRAS* and Serine/Threonine Kinase 11 (*STK11*) mutations is associated with the diverse phenotypic and heterogeneous oncogenic subtypes in the so-called “*KRAS* KL” NSCLC subgroup [11]. The KL co-mutation subgroup is designated from the K in *KRAS* and the L in liver kinase B1 (*LKB1*), also known as *STK11*. Unlike *KRAS*, *STK11* is a tumor suppressor gene and is a negative regulator of mammalian targets for rapamycin (mTOR) signaling [12]. Recent prospective and retrospective clinical studies have elucidated the potential prognostic and predictive implications of *KRAS* mutations and *KRAS* and *KRAS/STK11* co-mutations when treating patients with single or combined agents [13]. For example, the approval of immune checkpoint inhibitors (ICIs) has improved patient outcomes with metastatic NSCLC, regardless of their *KRAS* mutational status [14]. However, patients with *KRAS* pathogenic variants may have worse outcomes if they have co-occurring *KRAS/STK11* mutations [13].

Due to extensive mixed evidence, there needs to be a review of the current *KRAS* literature to better understand the potential clinical benefit of identifying prognostic and predictive biomarkers in metastatic NSCLC. As a point of reference, a prognostic biomarker indicates a patient’s survival, independent of the treatment received, because this biomarker is an indicator of innate tumor behavior. A predictive marker indicates therapeutic efficacy because there is an interaction between the biomarker and the therapy that affects a patient’s outcome. Over the past ten years, the KRAS treatment landscape has progressed from standard chemotherapy to more innovative approaches investigating the predictive value of ICIs and new *KRAS* G12C-targeted therapies. A good method for determining whether *KRAS* status is a predictive factor is looking at the outcomes of various cancer treatments in randomized controlled trials. Beyond clinical trials, the advent of “real world” multi-variate database analyses has allowed for many clinicians to investigate this topic across the treatment spectrum, early in first-line therapy, and in heavily pretreated NSCLC patients across various standards of care.

*KRAS*-mutated NSCLC is the most common type of oncogenic-driven lung cancer in Western populations. Most of these mutations occur in exons 2 and 3 [10]. A 15-pack-year smoking history increases the likelihood of a lung cancer diagnosis involving a *KRAS* mutation by six-fold compared to a never-smoker (*p* = 0.0001) [15]. Moreover, Calles et al. revealed that smokers with *KRAS*-mutant tumors were more likely to express programmed cell death ligand 1 (PD-L1) [16]. *KRAS* G12C is the most common *KRAS* pathogenic variant, occurring in 40–50% of NSCLC cases, and is a critical oncogenic driver found in ~13% of all lung adenocarcinomas [17]. Within *KRAS*, G12C transversion mutations are more common in current or former smokers, more common in women and reflect the primary mutagenic signature of the DNA damage caused by tobacco smoke [15]. In contrast, never-smokers have a higher frequency of G12D transition mutations [15].

The prognostic and predictive implications of *KRAS* mutations have been analyzed across various stages of lung cancer in multiple meta-analyses [14,18,19]. The current NCCN guidelines state that: “the presence of a *KRAS* mutation is prognostic of poor survival when compared to patients with tumors without *KRAS* mutations” [20]. The predictive implications of *KRAS* mutations as markers of outcomes to immuno-oncology agents, cytotoxic chemotherapy, and targeted therapy are of great interest to clinicians treating metastatic NSCLC. Most recently, sotorasib and adagrasib have garnered attention as the first two FDA-approved targeted agents for previously treated patients with *KRAS* G12C mutations in NSCLC [21,22].

*STK11* functions as a tumor suppressor gene. Located on chromosome 19, *STK11* encodes for Liver Kinase B1 (*LKB1*), an enzyme responsible for regulating cell metabolism, motility, differentiation, and metastasis [23]. *STK11* inactivation occurs via point mutations or deletions in ~10–30% of NSCLC cancers, ranking *STK11* as one of the most common mutated genes in lung adenocarcinoma after *TP53*, *KRAS*, and *EGFR* [24]. Somatic *STK11* mutations suppress programmed cell death, possibly leading to cell proliferation via an aberrant activation of the mTORC1 signaling pathway, which promotes cellular growth and tumorigenesis [25]. In addition, *STK11* mutations are associated with a reduced density of tumor-infiltrating cytotoxic CD8+ T lymphocytes and an increase in pro-inflammatory cytokine production, leading to a more immunosuppressive tumor microenvironment [13]. Supporting this observation of promoting a “cold tumor” microenvironment, several studies have demonstrated that *STK11* mutations are associated with a low or no PD-L1 expression and an accumulation of immunosuppressive cells and cytokines (i.e., IL-6) [26].

Unlike *KRAS*, the NCCN does not provide guidance on the prognostic value of *STK11* mutations in NSCLC. As ICI therapy has become the standard of care in metastatic NSCLC, this biomarker has started to become more relevant. Regarding their predictive implications for outcomes, various large ICI retrospective analyses have provided mixed results on *STK11* mutations [13,27].

*STK11* is the second most common co-mutated gene in patients with *KRAS*-mutant NSCLC, occurring in approximately 20–32% of metastatic NSCLC diseases [28,29]. The so-called “KL” subset of concurrent *KRAS–STK11* mutations defines a unique subset of NSCLC with an immunologically “cold” microenvironment and a more aggressive form of the disease. Often, *STK11* mutations correlate with *KRAS* activation and promote cell growth, as cells cannot halt the anabolic process during energy stress and metabolic depletion, leading to a potential increase in glutamine dependence [30].

This review highlights the clinical studies that have elucidated the potential prognostic and predictive implications of *KRAS*, *STK11*, and *KRAS/STK11* co-mutations when treating metastatic NSCLC patients across various treatments (i.e., chemotherapy and immune checkpoint inhibitors).

## 2. Materials and Methods

This critical review presents an analysis of the current literature specific to the prognostic and predictive implications of *KRAS* and *STK11* mutations and co-mutations, focusing on the outcomes for patients diagnosed with metastatic NSCLC. PubMed and the Web of Science were searched using the keywords: “*KRAS*”, “NSCLC”, “non-small-cell lung cancer”, “prognostic”, “predictive”, “co-occurring”, “computation”, “*STK11*”, and “*LKB1*”. Boolean operators were used to connect specific search keywords. Peer-reviewed reviews and meta-analyses published in English between 2016 and 2021 were included and duplicates were removed. Articles were excluded if they were solely pre-clinical in nature or focused on early-stage disease or diagnostics. Non-NSCLC- (i.e., small-cell), non-*KRAS-*, or non-*STK11*-focused studies were also excluded from this review.

## 3. Results and Description of Studies

Two hundred and three articles from PubMed were identified. In addition, 265 articles were identified from the Web of Science. Ninety duplicates were removed and 428 articles remained. All the exclusion criteria were applied, resulting in forty-four studies becoming a part of this critical review. Table 1 summarizes the study designs for the articles included in this critical review.

### 3.1. Prognostic Implications of KRAS Mutations in Metastatic NSCLC

Various published chemotherapy-based studies about the prognostic effect of *KRAS* mutations in metastatic NSCLC have produced mixed results. For example, one large meta-analysis of 13 studies with over 3000 metastatic NSCLC patients reported a significantly worse progression-free survival (PFS) with a hazard ratio (HR) of 1.3; *p* = <0.05 for *KRAS* mutant patients compared to those with *KRAS* wild-type (WT) after receiving first- or second-line platinum-based chemotherapy [19]. Similarly, in a much smaller analysis (N = 127 patients), the overall survival (OS) was significantly shorter for patients taking second- or third-line chemotherapy with *KRAS* mutations vs. *KRAS* WT (7.2 vs. 16.2 months, *p =* 0.008) [31].

In contrast to the aforementioned meta-analysis that reported a worse PFS for patients with *KRAS* mutations, a more extensive meta-analysis of 43 observational and randomized control trials (RCTs) revealed varied outcomes for patients with this oncogenic driver mutation [32]. The RCTs in this meta-analysis did not show an effect of the *KRAS* variants on OS. Conversely, the pooled multivariate analysis from the observational studies demonstrated a statistically significant negative prognostic effect of the mutant *KRAS* (HR, 1.71) [32]. Moreover, in a different meta-analysis evaluating the circulating tumor DNA (ctDNA) in 106 advanced NSCLC patients, the *KRAS* mutations that were detected signified a worse OS (HR, 2.07; *p* = <0.01) for the patients treated with chemotherapy [33].

Many studies did not show a prognostic difference across the various *KRAS* subtypes in NSCLC. When comparing the *KRAS* G12C vs. *KRAS* non-G12C mutation outcomes in NSCLC across various chemotherapies, there was no difference in the patients who received first-line platinum-based chemotherapy in three multi-variate *KRAS* mutant analyses [34,35,36]. In the US-based chart review (N = 218), the median OS was not significantly different among *KRAS* G12C vs. “all other *KRAS* mutants” (14.6 vs. 19.6 months, *p =* 0.43) for the patients who received first-line chemotherapy [34]. The second analysis (N = 346) demonstrated a similar OS among the *KRAS*-mutated and *KRAS*-WT patients (*p* = 0.54), with a similar OS compared to the *KRAS* G12C patients (*p* = 0.39) [35]. This analysis also revealed that the prevalence of brain metastases, a negative prognostic factor, was similar between the *KRAS*-mutant and *KRAS*-WT patients (33% vs. 40%, *p* = 0.17). However, a different analysis (N = 756) focusing on G12C vs. *KRAS* WT uncovered a numerically higher number of patients with brain metastasis (23.4% vs. 14.6%) [37]. In the smallest and third analyses of the *KRAS*-mutated patients treated with chemotherapy (N = 37), G12C *KRAS* mutations had no impact on their outcomes versus all the *KRAS* aberrations [36].

In contrast to the previous studies showing no effect on outcomes across the *KRAS* sub-types in NSCLC, two chemotherapy-based studies showed survival differences. One European analysis (N = 127) showed a worse overall survival for patients with G12C vs. other *KRAS* mutations (6.4 vs. 10.4 months, *p* = 0.011) [31]. When comparing the prognostic implications of *KRAS* G12D vs. G12C in a different study (N = 84), the investigators found that the survival of patients with *KRAS* G12D was shorter than that of patients with G12C variants after most patients received first- or second-line chemotherapy (*p* = 0.0001) [38].

Lung cancer therapies have advanced over the past ten years. The advent of immune checkpoint inhibitors (ICIs) and ICI combinations with chemotherapy has led to many multi-variate analyses analyzing the prognostic effect of various *KRAS* mutations in metastatic NSCLC and the predictive effects of the newer treatments. In one of the most extensive real-world *KRAS* G12C analyses (N = 743) ever conducted across multiple lines of therapy (67% of patients received ICI therapy), the OS for the NSCLC G12C sub-cohort was similar to that for all the NSCLC patients (15.2 vs. 14.8 months) [37]. In support of this notion of similar outcomes for metastatic patients with *KRAS* G12C or *KRAS* non-G12C mutations, the Arbour et al. analysis of comparable size (N = 770) demonstrated similar outcomes for *KRAS* G12C (13.4 months) vs. non-G12C patients (13.1 months, *p* = 0.96) across a heterogeneously treated population with either chemotherapy or ICI [39].

When looking exclusively at ICI monotherapy in a real-world registry via the IMMUNOTARGET study (N = 271), there was no significant PFS difference found between *KRAS* G12C vs. *KRAS* G12D or other *KRAS* mutations (*p* = 0.47) [40]. Interestingly, a PD-L1-positive expression and having a *KRAS* mutation were correlated with a longer PFS (7.2 vs. 3.9 months) (*p* = 0.01) [40]. Across a more heterogeneous prospective German registry of various treatments (N = 411) and a French multi-variate analysis (N = 162), where the majority of patients had received a combination of chemotherapy and ICI or ICI alone, no OS differences were found between *KRAS* WT, G12C, or non-G12C mutations [41]. *KRAS* mutational status was not found to be prognostic [41,42].

In contrast, a Dutch multi-variate analysis (N = 1357) demonstrated that *KRAS* mutations were associated with a decreased median survival compared to *KRAS* WT after chemotherapy: 19.4 vs. 24.7 months (HR, 1.25) [43]. Regarding *KRAS* G12D specifically, Aredo et al. (N = 186) revealed a negative prognostic effect with this mutation, as they found a significant association with a worse OS (HR, 2.43; *p* = 0.021) [44].

### 3.2. Predictive Effect of KRAS Mutations in Metastatic NSCLC

Several analyses have described whether one type of chemotherapy could lead to better outcomes for patients with *KRAS* mutations. When comparing combination chemotherapy in the first-line in one of the largest real-world multivariate analyses with *KRAS* mutations (N = 1190) to date, the investigators found that taxane-platinum combination therapy was associated with an improved time to treatment progression (TTP) (HR, 0.31; *p* < 0.001), especially in *KRAS* G13D patients (HR, 0.47; *p* = 0.05), and pemetrexed was associated with a worse TTP, particularly in *KRAS* G12V patients (HR, 0.55; *p* = 0.04) [45]. However, no impact on overall survival was found across the chemotherapy regimens and various types of *KRAS* mutations. In comparison to Renaud’s study, a significant difference in OS (9.7 versus 26.9 months: HR 1.93, *p* = 0.002) was found in patients who received first-line pemetrexed-based platinum doublet compared to those who received a non-pemetrexed-based platinum doublet in a retrospective analysis of 138 patients with *KRAS* mutations [46]. When looking at pemetrexed vs. gemcitabine in a different analysis (N = 334), the PFS HR for pemetrexed-treated patients with a G12C (N = 13) mutation, in comparison to subjects with *KRAS* WT was 1.96 (*p* = 0.045) [47]. However, other mutations failed to show significant clinical differences [47].

*KRAS*-mutation sub-groups analyses have been conducted with three FDA-approved ICIs across multiple lines of therapy, via monotherapy or in combination with chemotherapy, to help explore the predictive effect of *KRAS* variants in metastatic NSCLC [48,49,50]. Several multi-variate studies have demonstrated multiple outcomes as the treatment landscape has progressed beyond chemotherapy with single-agent ICI in pre-treated patients. In an extensive Italian Expanded Access program (N = 530), where nivolumab was primarily used in the second-/third-line, *KRAS* status was found not to be a reliable predictor of nivolumab efficacy, as there were no notable differences in the median OS of 11.2 vs. 10 months (*p =* 0.8) in *KRAS*-mutated vs. *KRAS*-wild-type patients (*p* = 0.86) [48]. A German analysis supported these findings (N = 176), showing that *KRAS*-mutated patients did not seem to obtain a PFS benefit from ICI vs. chemotherapy when receiving ICI in the second-line with post-chemo progression [49]. In contrast, an analysis from Greece (N = 265)) showed that pembrolizumab prolonged overall survival in comparison to any other treatment (*p* < 0.001), and this effect was more pronounced in the *KRAS*-mutated patients compared to those with *KRAS* WT [50]. Regarding PDL1 expression, one analysis (N = 282) showed that, in *KRAS*-mutant NSCLC, the efficacy of ICIs was higher, even though this was not statistically significant for patients with a PD-L1 expression >1% vs. <1%. This finding of ICI efficacy was especially true when the PD-L1 expression was high (50% PD-L1 expression) [42].

To explore the potential predictive value of *KRAS* in immune checkpoint inhibitors (ICI), Liu conducted a heterogeneous pooled analysis of 23 studies and 5326 patients, which showed that *KRAS*-mutated tumors were more likely to be programmed death-ligand 1 (PD-L1)-positive (*p* = 0.037) and have a higher tumor mutational burden (TMB) status than *KRAS* WT tumors (*p* = 0.0002) [51]. As PDL1 expression is a predictive biomarker of ICI, this was the first reported systematic study to reveal the superior efficacy of anti-PD-1/PD-L1 immunotherapy in *KRAS*-mutant NSCLC [51].

More specifically, analyses in two large, randomized phase 3 studies uncovered the predictive value of *KRAS* mutations and improvements in OS when using ICI compared to other standard-of-care treatments in patients with *KRAS* variants. The first meta-analysis, which included three randomized clinical trials (Checkmate-057 (N = 582), POPLAR (N = 287), and OAK (N = 850)), highlighted how ICIs significantly improved the overall survival in previously treated *KRAS* mutant NSCLC patients (HR, 0.64; *p* = 0.03) [52]. Interestingly, the second large meta-analysis of five clinical trials (N = 3025) using single-agent ICIs replicated an almost identical HR as compared to Kim’s analysis when comparing the OS in *KRAS*-mutated patients (HR, 0.65; *p* = 0.03) vs. *KRAS* WT patients [53]. Outside of these meta-analyses, a phase 2 single-arm atezolizumab (BIRCH) trial (N = 659) highlighted the predictive value of ICI in patients who were *KRAS* mutated vs. *KRAS* WT across different lines of therapy: not estimable vs. 20.1 months (first-line), 17.7 months vs. 15.1 months (second-line), and 12.1 months vs. 13.8 months (third-line or greater), respectively [54].

Alessi’s univariate analysis (N = 195) study showed no difference in the PFS or OS for *KRAS*-mutated vs. *KRAS*-WT patients with a >50% PDL1 expression [55]. Within Sun’s *KRAS* analysis of patients with a PDL1 expression greater than 50% that were treated with ICI monotherapy (N = 280), *KRAS* mutation was associated with a superior survival compared to *KRAS* WT (OS, 21.1 vs. 13.6 months, *p* = 0.03), and the OS did not differ between the patients with *KRAS* mutations treated with ICI monotherapy vs. triplet chemo-immunotherapy (OS, 21.1 vs. 20.0 months; HR, 1.03; *p* = 0.78) [56].

### 3.3. Predictive Implications of Other Treatments beyond Chemotherapy or ICI in KRAS-Mutated NSCLC

Several *KRAS*-mutant-focused clinical trials in clinical development have focused on tyrosine kinase inhibitors in combination with other agents and other novel mechanisms beyond chemotherapy or ICI. Major signaling pathways, including mitogen-activated protein kinase (MEK), often characterize mutant *KRAS* tumor proliferation and survival, so inhibiting their signaling mechanism is a valid approach for overcoming these pathways [57]. However, the majority of the agents studied did not target *KRAS* directly and did not predict clinical benefits. In a phase II clinical trial (N = 55), defactinib, a selective oral inhibitor of focal adhesion kinase (FAK), demonstrated modest clinical activity in heavily pre-treated *KRAS*-mutated NSCLC patients [70]. Focusing on the MAPK pathway in a larger phase III randomized study (N = 510), selumetinib (a MEK1 and MEK2 inhibitor) did not show an improvement in combination with docetaxel for the PFS (primary endpoint: 3.9 vs. 2.8 months, *p* = 0.44) or OS in pre-treated metastatic NSCLC patients [58]. Recent advances in CDK4/6 clinical development led to the phase III Juniper study. In this large, randomized trial (N = 453), abemaciclib (a potent and selective inhibitor of CDK 4 and 6) was compared to erlotinib (EGFR inhibitor) in *KRAS*-mutated patients who progressed after platinum-based chemotherapy and one additional therapy. Unfortunately, the primary endpoint of the OS was not reached: 7.4 months (abemaciclib) vs. 7.8 months with erlotinib (*p* = 0.77) [59]. In a smaller 1b clinical trial with abemaciclib in combination with pembrolizumab in *KRAS*-mutated patients (N = 50), the combination of abemaciclib and pembrolizumab resulted in a more significant toxicity compared to that previously reported for each individual treatment. This combination led to a higher discontinuation rate when compared to those of JUNIPER and KEYNOTE-042 (32% versus 12% and 9%, respectively) [60]. Regarding novel mechanisms, pelareorep (Dearing strain of reovirus serotype immune-oncolytic virus) was studied in the second-line in the randomized Canadian Cancer Trials Group (CCTG) IND211 trial (N = 166) [61]. Pelareorep did not improve the PFS in *KRAS*-mutated patients in a sub-group analysis [61].

Two agents that have gained FDA approval for metastatic NSCLC patients with *KRAS* G12C mutations are sotorasib and adagrasib [21,22]. In the phase 2 registrational (CodeBreaK100) trial (N = 126), sotorasib demonstrated a PFS of 6.8 months and an OS of 12.5 months in patients who were previously treated with standard therapies (90% previously treated with chemotherapy and ICI) [21]. The adagrasib approval was based on KRYSTAL-1, where its efficacy was evaluated in 112 patients: the median PFS was 6.5 months, and the median OS was 12.6 months [22].

### 3.4. Prognostic and Predictive Implications of STK11 Mutation in Metastatic NSCLC

In one of the most extensive *STK11* mutational analyses (N = 454) ever published, the authors concluded that “*STK11* mutations are prognostic and not predictive biomarkers for ICI therapy and should not be used to exclude patients from ICI therapy”, as there was no significant interaction between ICI and *STK11* mutations affecting OS [62]. In support of this conclusion, a second large, real-world study focusing on *STK11* in NSCLC showed worse OS outcomes in patients with *STK11* mutations (N = 328), regardless of treatment (chemotherapy or ICI therapy), versus *STK11* WT metastatic NSCLC [63].

Moreover, a meta-analysis including two atezolizumab clinical trials (N = 165) supported the OS improvement seen with ICI in patients with *STK11* mutations [64]. Coefficients from the multivariable logistic regression model were used to create prediction scores. The prediction scores for the OS in the *STK11*-mutated patients with atezolizumab vs. docetaxel resulted in a statistical difference (HR, 0.21, *p* = 0.04) [64]. In contrast, Skoulidis et al. concluded in 2018 that *STK11*/LKB1 genomic changes have a deleterious influence on the clinical responses to PD-1 inhibitors in PDL1-positive NSCLC, suggesting that a lack of response is partially independent of PD-L1 expression [13].

### 3.5. Prognostic Implications of STK11–KRAS Co-Mutations in Metastatic NSCLC

Beyond *STK11* mutations alone, the “KL” subset includes concurrent *KRAS–STK11* mutations. This subset defines a unique subset of NSCLC and a more aggressive form of the disease associated with a poorer prognosis and reduced survival [13]. This prognostic effect was further exemplified in two real-world analyses, where KL was associated with a significantly worse OS, regardless of therapy [62,63]. The first analysis of 37 *STK11*-mutated patients demonstrated the median overall survival of KL (7.1 months) vs. *STK11* alone (16.1 months) (*p* = 0.001) [66]. The second large analysis included 327 patients who had *STK11* mutations and were treated with either chemotherapy or ICI. When treated in the first-line with chemotherapy, patients’ OSs were as follows: KL 11.7 months vs. *STK11* mutated 11.7 months vs. *KRAS/STK11* WT 10.0 months [62]. ICI treatment in the first-line resulted in the respective OSs of: KL 10.0 months vs. *STK11* mutated 14.2 months vs. *KRAS-STK11* WT 18.2 months [62]. The authors concluded that *STK11–KRAS* co-mutations confer a poor prognosis in NSCLC, regardless of treatment class, and that these co-mutations were enriched for negative PD-L1 staining (75.8% vs. 60.8%, *p* < 0.001) [62]. In a large, “real-world” study (N = 7069) across many lines of treatment (first-, second-, or third-line of therapy), *KRAS* G12C/*STK11* co-mutations were not shown to impact the median OS as compared to *KRAS* WT or all NSCLC patients (including all *KRAS* mutations beyond G12C): 9 vs. 8.6 months, vs. 8.9 months in the patients [37]. With regard to incidence and OS, there was a significant association between the *STK11* (N = 25) and *KRAS* statuses, where mutations co-occurred 52% of the time (*p* = 0.008) in Facchinetti’s analysis, but the *STK11* status did not impact the OS in the multivariate analysis (N = 302) [66].

### 3.6. Predictive Implications of STK11–KRAS Co-Mutations in Metastatic NSCLC

Beyond randomized clinical trials, one real-world analysis (N = 95) demonstrated that *STK11/KRAS* co-mutations were not associated with an improved survival for patients who received nivolumab or pembrolizumab monotherapy (*STK11* (HR, 1.3; *p* = 0.22)) [67]. Arbour et al. determined that TMB was associated with a difference in the OS from the time of initiation of immunotherapy, as patients with co-mutations and a higher TMB were found to have a more prolonged survival from the start of treatment (HR, 0.9; *p* = 0.02) [67]. This is in stark contrast to a previous, widely cited analysis (N = 174) pointing to the negative predictive effect of *STK11* co-mutations as a major driver of primary resistance in relation to ICI [13]. Of note, there were significant differences in the response rates among the *KRAS/STK11* (KL) (7.4%) vs. *KRAS* (28.6%) NSCLC subgroups (*p* < 0.001) in the Stand Up To Cancer (SU2C) cohort (primarily nivolumab treatment) and patients treated with nivolumab in the phase III CheckMate-057 (RR: KL 0% vs. *KRAS* 18.2%, *p* = 0.04) [13]. The lower response rates and trends toward inferior outcomes with ICI in the KL subgroup are thought, in part, to be due to the effects of the LKB1 loss in the tumor microenvironment, despite the presence of an intermediate or high TMB [68]. In addition, other outcomes were significantly impacted in the KL NSCLC group, as these patients exhibited a shorter progression-free (*p* < 0.001) and overall (*p* = 0.0015) survival compared to those of the *KRAS* mutant/STK wild-type patients [13]. In support of these results, an extensive multi-institution, multi-variate, real-world analysis demonstrated that *STK11* co-mutations (N = 260) confer a worse OS after immunotherapy among patients with *KRAS* mutations but not among *KRAS* WT patients (HR, 2.09; *p* < 0.0001) [69].

Beyond ICI and chemotherapy, the newest *KRAS*-G12C-targeted therapies resulted in tumor reductions in patients with *KRAS-STK11* co-mutations. Sotorasib (a *KRAS* G12C inhibitor) demonstrated a 50% response rate (RR) for 11 out of 22 patients with co-*STK11* mutations (all the patients had *KRAS* G12C mutations) [21]. In addition, adagrasib showed a 64% RR for patients with concurrent *KRAS* and *STK11* mutations [22].

## 4. Discussion

Overall, identifying *KRAS* mutations in metastatic NSCLC provides some demonstrated prognostic value. *KRAS* aberrations in three meta-analyses pointed to a statistically significant worse PFS (HR, 1.3) and worse OS (HR, 1.71 and 2.07) [19,32,33]. The authors concluded that “*KRAS* mutation is a weak but valid predictor for poor prognosis.” [19]. The current NCCN guidelines state, with a more definitive stance: “the presence of a *KRAS* mutation is prognostic of poor survival when compared to patients with tumors without *KRAS* mutations” [20].

For patients who received first-line platinum-based chemotherapy, three multivariate analyses did not show a worse prognosis if they had a *KRAS* G12C variant vs. *KRAS* non-G12 variants [34,35,36]. The Svaton et al. analysis showed a worse prognosis for *KRAS* G12C patients than those with *KRAS* non-G12C (6.4 vs. 10.4 months, *p* = 0.011) [31]. When analyzing G12D vs. G12C specifically, Cai’s analysis of 84 patients with *KRAS* mutations demonstrated that the patients with *KRAS* G12D had a shorter OS than those with G12C variants (*p* = 0.0001) [38]. However, after controlling for several variables that lead to a worse prognosis (i.e., brain metastases), it might be that the chemotherapy of choice could have led to different outcomes vs. categorizing all chemotherapies as equal in the realm of *KRAS* mutations [31,38].

In three retrospective studies, chemotherapy selection was possibly predictive of outcomes (taxane vs. pemetrexed vs. gemcitabine) when looking across various *KRAS* mutations [45,46,47]. In one of the most extensive *KRAS* mutation analyses to date (N = 1190), Renaud et al. found that taxane-platinum combination therapy was associated with an improved Time to Treatment Progression (TTP) HR, 0.31; *p* < 0.001) vs. pemetrexed, especially in *KRAS* G13D patients (HR, 0.47; *p* = 0.05), and pemetrexed was associated with a worse TTP, particularly in *KRAS* G12V patients (HR, 0.55; *p* = 0.04) [45]. In comparison to Renaud’s study, Ricciuti et al.’s study (N = 356) demonstrated a significant overall survival difference (OS 9.7 vs. 26.9 months, HR 1.93, *p* = 0.002) in patients who received first-line pemetrexed-based platinum doublet compared to those who received a non-pemetrexed-based platinum doublet in a retrospective analysis of 138 patients with *KRAS* mutations [46]. In Park’s study, the PFS was worse (HR:1.96, *p* = 0.045), with no difference in the OS for pemetrexed-treated subjects with G12C mutations compared to subjects with *KRAS* WT, but other mutations failed to show clinical significance [47].

When treating patients with *KRAS* aberrations using ICI, there are mixed results regarding prognoses. In one of the largest real-world *KRAS* G12C analyses ever conducted (N = 743), the overall survival for the NSCLC G12C sub-cohort (15.2 vs. 14.8 months) was similar compared to a cohort that included all NSCLC patients (N = 7069), regardless of driver mutation status and irrespective of the type of therapy used [37]. When looking across several meta-analyses that contained large phase III randomized studies (Checkmate-57, POPLAR, and OAK) on pre-treated patients, there was a clear predictive benefit with ICI vs. chemotherapy with regard to OS and patients with *KRAS*-mutated NSCLC (HR. 64, *p* = 0.03) [52,53].

Two important analyses from landmark studies support this notion of a *KRAS*-predictive effect. The KEYNOTE-042 study demonstrated a predictive effect. In this study, the first-line use of pembrolizumab monotherapy for patients with a PDL1 of > 1% and *KRAS* G12C mutations showed an improvement in their response rates (66.7% vs. 23.5%) and OS (28 vs. 11 months) compared to chemotherapy, but did not show an improvement among *KRAS* WT patients (OS 15 vs. 12 months with chemotherapy) [71]. The KEYNOTE-189 exploratory analysis demonstrated that triplet pembrolizumab-pemetrexed-platinum (irrespective of PD-L1 expression) therapy “should be considered as a standard first-line treatment for patients with metastatic non-squamous NSCLC, regardless of *KRAS* mutation status” [72]. There was a numerical improvement in the *KRAS*-mutation OS: triplet chemo-ICI therapy 21 months vs. 14 months with the chemotherapy doublet (HR, 0.79, (0.45–1.38)) and a statically significant survival benefit among *KRAS*-mutated patients (PFS 9 versus 5 months, HR, 0.47) [72]. When addressing a higher >50% PDL-1 cutoff, Sun et al. found a higher OS in patients treated with ICI with *KRAS* mutations vs. those with *KRAS* wild type (21.1 vs. 13.6 months, *p* = 0.03) [56].

Looking across various *KRAS* variants, the IMMUNOTARGET study found no significant PFS difference between G12 aberrations with single-agent ICI, as demonstrated with G12C vs. G12D or other *KRAS* mutations (*p* = 0.47) [40]). Regarding *KRAS* G12D specifically, Aredo et al. revealed a negative prognostic effect with these mutations, as they found a significant association with a worse OS (HR, 2.43; *p* = 0.021) [44]. Furthering the study’s analysis, a PD-L1-positive expression and positive *KRAS* status were correlated with a longer PFS (7.2 vs. 3.9 months, *p* = 0.01) [40]. Across a more heterogeneous population, no OS differences were found between patients with *KRAS* WT, G12C, or non-G12C mutations. In contrast, one multi-variate analysis (N = 392) demonstrated that *KRAS* mutations were associated with a decreased median survival in comparison to *KRAS* WT after first-line treatment: 19.4 vs. 24.7 months (HR, 1.25) [43].

Based on several studies, patients with *STK11* mutations prognostically had a worse OS, regardless of treatment, as demonstrated in the KEYNOTE-189 analysis: (10.6 months vs. 16.7 months, HR: 1.58, *p* = 0.008) [62,73]. In the updated KEYNOTE-189 analysis, the pembrolizumab chemo-platinum triplet was associated with numerically better outcomes than standard chemotherapy, regardless of *STK11* mutation status (HR: 0.75 (0.37–1.50)): *STK11* mutation: OS 17 months (triplet chemotherapy-ICI) vs. 8 months (standard chemotherapy) and *STK11* WT: 23 months vs. 12 months [73]. Interestingly, the PD-L1 Tumor Proportion Score (TPS) tended to be lower in the patients with vs. without *STK11* mutations (0% vs. 15%), and the TMB score tended to be higher in patients with *STK11* mutations (209 vs. 146) [73].

In one of the most extensive *STK11* mutational analyses (N = 454) ever published, there was no significant predictive interaction between ICI treatment and *STK11* mutations on OS [63]. The authors concluded that *STK11* mutations are prognostic and not predictive biomarkers for ICI therapy and should not be used to exclude patients from ICI therapy. [63]. In contrast, the KEYNOTE-042 exploratory analysis with single-agent pembrolizumab was associated with a better OS in patients with *STK11* mutations vs. without and had better outcomes than chemotherapy: 18 vs. 8 months (HR, 0.75) [74].

Patients with co-occurring *KRAS* and *STK11* co-mutations comprise a unique subset with a more aggressive form of NSCLC, which points to a major driver of primary resistance, more so than *KRAS* mutations or *STK11* alone [13]. For example, in Skoulidis et al.’s analysis, there was a significant predictive effect on the response rate (*p* = 0.001 & 0.04) when comparing KL vs. *KRAS*-mutated patients in Checkmate-057 (7.4% vs. 28.6) and SU2C (0% vs. 18.2%) [13]. In support of these results, Ricciuti’s real-world multi-variate analysis demonstrated that *KRAS*/*STK11* co-mutations predict a worse OS than *KRAS*-mutated-*STK11* WT (4.4 months vs. 20.8 months, *p* < 0.0001) [69]. In contrast to Skoulidis and Papillon-Cavanaugh, Spira demonstrated that *KRAS* G12C/*STK11* specifically did not impact the median OS compared to the general NSCLC lung population [37]. More recently, when looking at ICI triplet therapy vs. chemo-doublet therapy in first-line *STK11*-mutated patients (non-*KRAS* mutated), the triplet did not improve the PFS (mPFS 4.8 m vs. 4.3 m, *p* = 0.75) or OS (mOS 10.6 m vs. 10.3 m, *p =* 0.79) [75]. The response rates with triplet ICI differed significantly between the two groups (*STK11* mutated 32.6% vs. *STK11* WT 44.7%, *p* = 0.04) compared to that with chemotherapy-platinum alone [75].

Several trials focusing on mechanisms beyond chemotherapy or ICI did not provide any survival benefit for patients with *KRAS* mutations. Defactinib, selumetinib, pelareop, and abemaciclib in combination with pembrolizumab, did not result in a survival benefit for patients with *KRAS* mutations [58,59,61,70]. The FDA approved sotorasib as the first targeted agent for patients with *KRAS* G12C mutations, as a result of the phase 2 CodeBreaK 100 trial [21]. More recently, in a large, randomized phase 3 study, the CodeBreaK 200 resulted in a PFS advantage (primary endpoint) of sotarasib vs. docetaxel (5.6 vs. 4.5 months) (*p* < 0.001), but this did not translate into an OS benefit (secondary endpoint: HR, 1.01) [76]. In addition, adagrasib was the second *KRAS* G12C approved by the FDA as a result of the KRYSTAL-1 study, where patients with *KRAS* G12C mutations had an ORR of 43% and a median duration of response (DOR) of 8.5 months [77].

There are two apparent limitations of this critical review. There was a single reviewer, increasing the risk of bias, and the dates of the articles were from 2016–2021.

## 5. Conclusions

The path to precision medicine has increased the use of NGS testing and improved the detection of *KRAS* and *STK11* mutations. *KRAS* mutations are the most frequently reported gain-of-function oncogenic driver mutations in NSCLC. *KRAS* mutations are found in 30% of NSCLC adenocarcinomas, while patients with *KRAS* mutations are *KRAS*/*STK11* co-mutated 15–32% of the time in the metastatic setting. *KRAS* mutations are associated with poor prognoses and have been determined to be a valid but weak prognostic biomarker among patients diagnosed with NSCLC. *KRAS* mutations in NSCLC have mixed results as predictive clinical biomarkers for immune checkpoint inhibitors treatment. *STK11* mutations are prognostic and had mixed results as predictive biomarkers for ICI therapy in the studies included in this review. However, *KRAS*/*STK11* co-mutations may predict a primary resistance to ICI. Several randomized trials focusing on mechanisms beyond chemotherapy or ICI did not result in any significant overall survival benefit for patients with *KRAS* mutations. The vast majority of investigators in this review conducted retrospective multi-variate analyses when looking at either *KRAS*, *STK11*, or *KRAS/STK11* co-mutations. Prospective *KRAS/STK11* biomarker-driven randomized trials are needed to assess the predictive effect of these mutations on the overall survival for patients with metastatic NSCLC.

## Figures and Tables

**Table 1:** Summary of study designs for articles included in this critical review.

Study Title	Study Method/ Design	Line of Therapy	Treatment Arms	Biomarker	N	Author/ Reference	Year of Publication
The prognostic role of *KRAS* mutation in patients with advanced NSCLC treated with second- or third-line chemotherapy	Single-center, retrospective medical record review	2nd- or 3rd-line	Chemotherapy: Pemetrexed or docetaxel	*KRAS* mutation	39	Svaton et al. [31]	2016
*KRAS* mutation as a prognostic factor and predictive factor in advanced/metastatic non-small cell lung cancer: a systematic literature review and meta-analysis	Systematic literature review and meta-analysis (43 studies)	1st-, 2nd-, 3rd-, or 4th-line	Various (including chemotherapy and EGFR inhibitors)	*KRAS* mutation	701	Goulding et al. [32]	2020
Prognostic value of *EGFR* and *KRAS* in circulating tumor DNA in patients with advanced non-small cell lung cancer: a systematic review and meta-analysis	Systematic literature review and meta-analysis (13 studies)	1st-line	Chemotherapy	*KRAS* mutation	104	Fan et al. [33]	2017
First-line chemotherapy responsiveness and patterns of metastatic spread identify clinical syndromes present within advanced *KRAS* mutant non-small-cell lung cancer with different prognostic significance	Multi-center, retrospective medical record review	1st-line	Chemotherapy	*KRAS* mutation	218	Iams et al. [34]	2018
Real-world outcomes in *KRAS* G12C mutation-positive non-small cell lung cancer	Single-center, prospective Thoracic Malignancies Cohort (TMC)	1st-, 2nd-, or 3rd-line	Chemotherapy +/− ICI (primarily chemo in 1st line) Single-agent ICI (primarily in 2nd-line)	*KRAS* mutation	144	Cui et al. [35]	2020
Concomitant genomic alterations in *KRAS* mutant advanced lung adenocarcinoma	Multi-center, multivariate analysis	1st-line	Chemotherapy	*KRAS* mutation	37	Gibert et al. [36]	2020
A retrospective observational study of the natural history of advanced non-small-cell lung cancer in patients with *KRAS* p.G12C mutated or wild-type disease	Multi-center (280 sites), retrospective observational cohort study	1st-, 2nd-, or 3rd-line	Chemotherapy with or without ICI (67%) ICI	*KRAS* mutation	743	Spira et al. [37]	2021
The prevalence and prognostic value of *KRAS* co-mutation subtypes in Chinese advanced non-small cell lung cancer patients	Single-center, retrospective observational cohort	1st-line	Chemotherapy: Pemetrexed-platinum	*KRAS* mutation	84	Cai et al. [38]	2020
Treatment outcomes and clinical characteristics of patients with *KRAS*-G12C-mutant non-small cell lung cancer	Single-center, retrospective, medical record review	Primarily 1st- or 2nd-line	(49%) Platinum-doublet chemo (1L or 2L) (46%) ICI (1L or 2L) (39%) Single-agent PD-L1 inhibitor (7%) Chemo combined with ICI (1L)	*KRAS* mutation	1194	Arbour et al. [39]	2021
Immune checkpoint inhibitors for patients with advanced lung cancer and oncogenic driver alterations: results from the IMMUNOTARGET registry	Global multi-center registry	1st–5th-line	Single-agent ICI	*KRAS* mutation	271	Mazieres et al. [40]	2019
*KRAS* G12C-mutated advanced non-small cell lung cancer: a real-world cohort from the German prospective, observational, nationwide CRISP Registry (AIO-TRK-0315)	Multi-center (98 sites), prospective, observational registry	1st-, 2nd-, or 3rd-line	(55%) Platinum doublet chemotherapy and (45%) Chemo +/− ICI (1L) (60%) ICI +/− chemo and (40%) chemo (2L)	*KRAS* mutation	411	Sebastian et al. [41]	2021
Efficacy of immune checkpoint Inhibitors in *KRAS*-mutant non-small cell lung cancer (NSCLC)	Multi-center (two sites), retrospective, medical record review	1st–6th-line. Primarily 2nd- or 3rd-line: 77% of patients	Primarily 2nd- or 3rd-line single agent ICI (88%) Nivolumab	*KRAS* mutation	162	Jeanson et al. [42]	2019
Programmed cell death ligand-1 expression and survival in a cohort of patients with non-small cell lung cancer receiving first-line through third-line therapy in Denmark	Danish Lung Cancer Registry, medical record review	1st-, 2nd- or 3rd-line	Chemotherapy	*KRAS* mutation	385	Hedgeman et al. [43]	2021
Impact of *KRAS* mutation subtype and concurrent pathogenic mutations on non-small cell lung cancer outcomes	Single-center, retrospective medical record review	NA	Chemotherapy, ICI	*KRAS* mutation *KRAS -STK11* co-mutation	202 11	Aredo et al. [44]	2019
*KRAS*-specific amino acid substitutions are associated with different responses to chemotherapy in advanced non-small-cell lung cancer	Single-center, retrospective, medical record review	1st-line	Chemotherapy	*KRAS* mutation	1190	Renaud et al. [45]	2018
Clinical outcomes to pemetrexed-based versus non-pemetrexed-based platinum doublets in patients with *KRAS*-mutant advanced non-squamous non-small cell lung cancer	Multi-center, retrospective, medical record review	1st-line	Chemotherapy-platinum	*KRAS* mutation	138	Ricciuti et al. [46]	2020
*KRAS* G12C mutation as a poor prognostic marker of pemetrexed treatment in non-small cell lung cancer	Single-center, retrospective, medical record review	1st-, 2nd-, or 3rd-line	Chemotherapy	*KRAS* mutation	45	Park et al. [47]	2017
Efficacy of nivolumab in pre-treated non-small-cell lung cancer patients harboring *KRAS* mutations	Multi-center (168 centers), retrospective, medical record review. Expanded access program (EAP)	2nd-, 3rd-, or 4th-line	Nivolumab	*KRAS* mutation	206	Passiglia et al. [48]	2019
Immune checkpoint inhibitors versus second-line chemotherapy for patients with lung cancer refractory to first-line chemotherapy	Multi-center, retrospective, medical record review	2nd-line	ICI Chemotherapy	*KRAS* mutation	52	Lefebvre et al. [49]	2020
Association between PD-L1 expression and driver gene mutations in non-small cell lung cancer patients: correlation with clinical data	Single center, retrospective, medical record review	1st-, 2nd-, or 3rd-line	ICI (39%) Pembrolizumab single agent (1L, 2L, or 3L) Chemotherapy	*KRAS* mutation	62	Karatrasoglou et al. [50]	2020
The superior efficacy of anti-PD-1/PD-L1 immunotherapy in *KRAS*-mutant non-small cell lung cancer that correlates with an inflammatory phenotype and increased immunogenicity	Meta-analysis (23 studies)	1st-, 2nd-, or 3rd-line	Various ICI Chemotherapy	*KRAS* mutation (first pooled analysis)	1244	Liu et al. [51]	2019
Prognostic value of *KRAS* mutation in advanced non-small-cell lung cancer treated with immune checkpoint inhibitors: a meta-analysis and review	Meta-analysis: (three trials: Checkmate-057, POPLAR, OAK)	2nd- or 3rd-line	Atezolizumab Nivolumab Docetaxel	*KRAS* mutation	148	Kim et al. [52]	2017
Clinical and molecular characteristics associated with survival among patients treated with checkpoint inhibitors for advanced non-small cell lung carcinoma: a systematic review and meta-analysis	Systemic review and meta-analysis (five randomized trials)	2nd-line (primarily)	Nivolumab, Pembrolizumab Atezolizumab Docetaxel	*KRAS* mutation	148	Lee et al. [53]	2018
Phase II trial of atezolizumab as first-line or subsequent therapy for patients with programmed death-ligand 1-selected advanced non-small-cell lung cancer (BIRCH)	Phase II, single-arm, prospective clinical trial	1st-, 2nd-, or 3rd-line	Atezolizumab	*KRAS* mutation	137	Peters et al. [54]	2017
Outcomes to first-line pembrolizumab in patients with PD-L1-high (≥50%) non-small cell lung cancer and a poor performance status	Multi-center (three centers), retrospective, medical record review	1st-line	Pembrolizumab	*KRAS* mutation	81	Alessi et al. [55]	2020
Association between *KRAS* variant status and outcomes with first-line immune-checkpoint-inhibitor-based therapy in patients with advanced non-small-cell lung cancer	Multi-center (280 centers), retrospective medical record review via Flatiron Health database	1st-line	ICI	*KRAS* mutation	573	Sun et al. [56]	2021
Phase II study of the focal adhesion kinase inhibitor defactinib (VS-6063) in previously treated advanced *KRAS* mutant non-small cell lung cancer	Phase II, prospective clinical trial	Median number of prior lines of therapy was four (range 1–8)	Defactinib (Focal Adhesion Kinase Inhibitor)	*KRAS* mutation	55	Gerber et al. [57]	2019
Selumetinib plus docetaxel compared with docetaxel alone and progression-free survival in patients with *KRAS*-Mutant advanced non-small cell lung cancer: the SELECT-1 randomized clinical trial	Multi-national (202 sites), 25 countries, randomized phase II, prospective trial	2nd-line	Selumetinib + docetaxel Docetaxel	*KRAS* mutation	510	Janne et al. [58]	2017
A randomized phase III study of Abemaciclib versus Erlotinib in patients with stage IV non-small cell lung cancer with a detectable *KRAS* mutation who failed prior platinum-based therapy: JUNIPER	Randomized phase III, multi-center, open-label	2nd- or 3rd-line	Abemaciclib Erlotinib	*KRAS* mutation	453	Goldman et al. [59]	2020
Abemaciclib in combination with pembrolizumab for stage IV *KRAS*-mutant or squamous NSCLC: a Phase 1b study	Multi-center, nonrandomized, open-label, phase 1b	1st-line	Abemaciclib in combination with pembrolizumab	*KRAS* mutation	25	Pujol et al. [60]	2021
Canadian Cancer Trials Group (CCTG) IND211: a randomized trial of pelareorep (Reolysin) in patients with previously treated advanced or metastatic non-small cell lung cancer receiving standard salvage therapy	Multi-center, randomized, phase II clinical trial	2nd-line	Chemotherapy +/− Pelareorep	NA	NA	Bradbury et al. [61]	2018
Sotorasib for lung cancers with *KRAS* p.G12C mutation	Multi-center, Phase I, clinical trial	2nd-line or greater	Sotorasib	*KRAS* mutation *KRAS-STK11* co-mutation	126 35	Skoulidis et al. [21]	2021
Targeting *KRAS* in non-small-cell lung cancer: Recent progress and new approaches	Review that included multiple studies (including Phase I Krystal-1)	2nd-line or greater	Adagrasib	*KRAS* mutation *KRAS-STK11* co-mutation	116 30	Reck et al. [22]	2021
*STK11 (LKB1)* mutations in metastatic NSCLC: Prognostic value in the real world.	Retrospective cohort study, medical record review via Flatiron Health database	1st- or 2nd-line	ICI (1L or 2L) Chemotherapy 1L	*STK11* mutation *KRAS-STK11* co-mutation	327 156	Shire et al. [62]	2020
*STK11* and *KEAP1* mutations as prognostic biomarkers in an observational real-world lung adenocarcinoma cohort	Retrospective cohort study, medical record review via Flatiron Health database	1st-line	(37%) Platinum-based chemotherapy combinations (25%) PD-1/PD-L1–based therapies (21%) Anti-VEGF–based therapies (14%) EGFR TKIs (4%) Single-agent chemotherapy	*KRAS* mutation *STK11* mutation	263 454	Papillon-Cavanaugh et al. [63]	2020
Atezolizumab prolongs overall survival over docetaxel in advanced non-small-cell lung cancer patients harboring *STK11* or *KEAP1* mutation	Retrospective analysis of OAK and POPLAR trials and single-center retrospective	(74%) 2nd-line (26%) 3rd-line	Atezolizumab Docetaxel	*STK11* mutation	38	Nie et al. [64]	2021
*STK11/LKB1* mutations and PD-1 inhibitor resistance in *KRAS*-mutant lung adenocarcinoma	Retrospective analysis of SU2C cohort and Checkmate-057	2nd-line (Checkmate-057)	(78%) Nivolumab	*KRAS*-mutation *KRAS-STK11* co-mutation	218 60	Skoulidis et al. [13]	2018
Impact of *KRAS* and *TP53* co-Mutations on outcomes after first-line systemic therapy among patients with *STK11*-mutated advanced non-small-cell lung cancer	Single-center retrospective medical record review	1st-line	Chemotherapy	*STK11* mutation *KRAS-STK11* co-mutation	18 19	Bange et al. [65]	2019
*LKB1/STK11* mutations in non-small cell lung cancer patients: descriptive analysis and prognostic value	Retrospective analysis of the MOSCATO and MSN (NCT02105168) studies	1st- or 2nd-line (72%) 1st-line	(72%) Chemotherapy	*STK11* mutation *KRAS-STK11* co-mutation	13 12	Facchinetti et al. [66]	2017
Effects of co-occurring genomic alterations on outcomes in patients with *KRAS*-mutant non-small cell lung cancer	Single-center retrospective medical record review	1st- or 2nd-line	Chemotherapy Nivolumab or pembrolizumab	*KRAS* mutation *KRAS-STK11* co-mutation	330 95	Arbour et al. [67]	2018
*TP53*, *STK11*, and *EGFR* mutations predict tumor immune profile and the response to anti-PD-1 in lung adenocarcinoma	Single-center retrospective medical record review	1st- or 2nd-line	Nivolumab or pembrolizumab	*KRAS* mutation *KRAS-STK11* co-mutation	11 6	Biton et al. [68]	2018
Diminished efficacy of programmed death-(Ligand)1 inhibition in *STK11*- and *KEAP1*-mutant lung adenocarcinoma is affected by *KRAS* mutation status	Multi-center retrospective medical record review	1st- or 2nd-line	(31%) ICI 1L (69%) IC1 2L	*KRAS* mutation *STK11* mutation	536 260	Ricciuti et al. [69]	2021

1L, 1st line; 2L, 2nd Line; ICI, Immune Checkpoint Inhibitor; VEGF, Vascular endothelial growth factor; chemo, chemotherapy; PD1, Programmed Cell Death; and PDL-1, Programmed Cell Death Ligand-1.

## Data Availability

Not applicable.

## References

[1] Thandra K.C., Barsouk A., Saginala K., Aluru J.S., Barsouk A. (2021). Epidemiology of lung cancer. Contemp. Oncol. (Pozn.).

[2] Cancer Stat Facts: Lung and Bronchus Cancer. https://seer.cancer.gov/statfacts/html/lungb.html.

[3] Siegel R.L., Miller K.D., Fuchs H.E., Jemal A. (2021). Cancer Statistics. CA Cancer J. Clin..

[4] Meza R., Meernik C., Jeon J., Cote M.L. (2015). Lung cancer incidence trends by gender, race and histology in the United States, 1973–2010. PLoS ONE.

[5] Soria J.C., Ohe Y., Vansteenkiste J., Reungwetwattana T., Chewaskulyong B., Lee K.H., Dechaphunkul A., Imamura F., Nogami N., Kurata T. (2018). Osimertinib in Untreated EGFR-Mutated Advanced Non-Small-Cell Lung Cancer. N. Engl. J. Med..

[6] Cox A.D., Fesik S.W., Kimmelman A.C., Luo J., Der C.J. (2014). Drugging the undruggable RAS: Mission possible?. Nat. Rev. Drug Discov..

[7] Mascaux C., Iannino N., Martin B., Paesmans M., Berghmans T., Dusart M., Haller A., Lothaire P., Meert A.P., Noel S. (2005). The role of RAS oncogene in survival of patients with lung cancer: A systematic review of the literature with meta-analysis. Br. J. Cancer.

[8] Blumenschein G.R., Smit E.F., Planchard D., Kim D.W., Cadranel J., De Pas T., Dunphy F., Udud K., Ahn M.J., Hanna N.H. (2015). A randomized phase II study of the MEK1/MEK2 inhibitor trametinib (GSK1120212) compared with docetaxel in KRAS-mutant advanced non-small-cell lung cancer (NSCLC)†. Ann. Oncol..

[9] Ettinger D.S., Wood D.E., Akerley W., Bazhenova L.A., Borghaei H., Camidge D.R., Cheney R.T., Chirieac L.R., D’Amico T.A., Dilling T.J. (2016). NCCN Guidelines Insights: Non-Small Cell Lung Cancer, Version 4.2016. J. Natl. Compr. Cancer Netw..

[10] Kris M.G., Johnson B.E., Berry L.D., Kwiatkowski D.J., Iafrate A.J., Wistuba I.I., Varella-Garcia M., Franklin W.A., Aronson S.L., Su P.F. (2014). Using multiplexed assays of oncogenic drivers in lung cancers to select targeted drugs. JAMA.

[11] Skoulidis F., Byers L.A., Diao L., Papadimitrakopoulou V.A., Tong P., Izzo J., Behrens C., Kadara H., Parra E.R., Canales J.R. (2015). Co-occurring genomic alterations define major subsets of KRAS-mutant lung adenocarcinoma with distinct biology, immune profiles, and therapeutic vulnerabilities. Cancer Discov..

[12] Zhou W., Marcus A.I., Vertino P.M. (2013). Dysregulation of mTOR activity through LKB1 inactivation. Chin. J. Cancer.

[13] Skoulidis F., Goldberg M.E., Greenawalt D.M., Hellmann M.D., Awad M.M., Gainor J.F., Schrock A.B., Hartmaier R.J., Trabucco S.E., Gay L. (2018). STK11/LKB1 Mutations and PD-1 Inhibitor Resistance in KRAS-Mutant Lung Adenocarcinoma. Cancer Discov..

[14] Borghaei H., Paz-Ares L., Horn L., Spigel D.R., Steins M., Ready N.E., Chow L.Q., Vokes E.E., Felip E., Holgado E. (2015). Nivolumab versus Docetaxel in Advanced Nonsquamous Non-Small-Cell Lung Cancer. N. Engl. J. Med..

[15] Dogan S., Shen R., Ang D.C., Johnson M.L., D’Angelo S.P., Paik P.K., Brzostowski E.B., Riely G.J., Kris M.G., Zakowski M.F. (2012). Molecular epidemiology of EGFR and KRAS mutations in 3,026 lung adenocarcinomas: Higher susceptibility of women to smoking-related KRAS-mutant cancers. Clin. Cancer Res..

[16] Calles A., Liao X., Sholl L.M., Rodig S.J., Freeman G.J., Butaney M., Lydon C., Dahlberg S.E., Hodi F.S., Oxnard G.R. (2015). Expression of PD-1 and Its Ligands, PD-L1 and PD-L2, in Smokers and Never Smokers with KRAS-Mutant Lung Cancer. J. Thorac. Oncol..

[17] Biernacka A., Tsongalis P.D., Peterson J.D., de Abreu F.B., Black C.C., Gutmann E.J., Liu X., Tafe L.J., Amos C.I., Tsongalis G.J. (2016). The potential utility of re-mining results of somatic mutation testing: KRAS status in lung adenocarcinoma. Cancer Genet..

[18] Román M., Baraibar I., López I., Nadal E., Rolfo C., Vicent S., Gil-Bazo I. (2018). KRAS oncogene in non-small cell lung cancer: Clinical perspectives on the treatment of an old target. Mol. Cancer.

[19] Pan W., Yang Y., Zhu H., Zhang Y., Zhou R., Sun X. (2016). KRAS mutation is a weak, but valid predictor for poor prognosis and treatment outcomes in NSCLC: A meta-analysis of 41 studies. Oncotarget.

[20] Ettinger D.S., Wood D.E., Aisner D.L., Akerley W., Bauman J.R., Bharat A., Bruno D.S., Chang J.Y., Chirieac L.R., D’Amico T.A. (2022). Non-Small Cell Lung Cancer, Version 3.2022, NCCN Clinical Practice Guidelines in Oncology. J. Natl. Compr. Cancer Netw..

[21] Skoulidis F., Li B.T., Dy G.K., Price T.J., Falchook G.S., Wolf J., Italiano A., Schuler M., Borghaei H., Barlesi F. (2021). Sotorasib for Lung Cancers with KRAS p.G12C Mutation. N. Engl. J. Med..

[22] Reck M., Carbone D.P., Garassino M., Barlesi F. (2021). Targeting KRAS in non-small-cell lung cancer: Recent progress and new approaches. Ann. Oncol..

[23] Larsen J.E., Minna J.D. (2011). Molecular biology of lung cancer: Clinical implications. Clin. Chest Med..

[24] Ding L., Getz G., Wheeler D.A., Mardis E.R., McLellan M.D., Cibulskis K., Sougnez C., Greulich H., Muzny D.M., Morgan M.B. (2008). Somatic mutations affect key pathways in lung adenocarcinoma. Nature.

[25] Alessi D.R., Sakamoto K., Bayascas J.R. (2006). LKB1-dependent signaling pathways. Annu. Rev. Biochem..

[26] Koyama S., Akbay E.A., Li Y.Y., Aref A.R., Skoulidis F., Herter-Sprie G.S., Buczkowski K.A., Liu Y., Awad M.M., Denning W.L. (2016). STK11/LKB1 Deficiency Promotes Neutrophil Recruitment and Proinflammatory Cytokine Production to Suppress T-cell Activity in the Lung Tumor Microenvironment. Cancer Res..

[27] Pons-Tostivint E., Lugat A., Fontenau J.-F., Denis M.G., Bennouna J. (2021). STK11/LKB1 Modulation of the Immune Response in Lung Cancer: From Biology to Therapeutic Impact. Cells.

[28] Scheffler M., Ihle M.A., Hein R., Merkelbach-Bruse S., Scheel A.H., Siemanowski J., Brägelmann J., Kron A., Abedpour N., Ueckeroth F. (2019). K-ras Mutation Subtypes in NSCLC and Associated Co-occurring Mutations in Other Oncogenic Pathways. J. Thorac. Oncol..

[29] Adderley H., Blackhall F.H., Lindsay C.R. (2019). KRAS-mutant non-small cell lung cancer: Converging small molecules and immune checkpoint inhibition. EBioMedicine.

[30] Galan-Cobo A., Sitthideatphaiboon P., Qu X., Poteete A., Pisegna M.A., Tong P., Chen P.H., Boroughs L.K., Rodriguez M.L.M., Zhang W. (2019). LKB1 and KEAP1/NRF2 Pathways Cooperatively Promote Metabolic Reprogramming with Enhanced Glutamine Dependence in KRAS-Mutant Lung Adenocarcinoma. Cancer Res..

[31] Svaton M., Fiala O., Pesek M., Bortlicek Z., Minarik M., Benesova L., Topolcan O. (2016). The Prognostic Role of KRAS Mutation in Patients with Advanced NSCLC Treated with Second- or Third-line Chemotherapy. Anticancer Res..

[32] Goulding R.E., Chenoweth M., Carter G.C., Boye M.E., Sheffield K.M., John W.J., Leusch M.S., Muehlenbein C.E., Li L., Jen M.H. (2020). KRAS mutation as a prognostic factor and predictive factor in advanced/metastatic non-small cell lung cancer: A systematic literature review and meta-analysis. Cancer Treat. Res. Commun..

[33] Fan G., Zhang K., Ding J., Li J. (2017). Prognostic value of EGFR and KRAS in circulating tumor DNA in patients with advanced non-small cell lung cancer: A systematic review and meta-analysis. Oncotarget.

[34] Iams W.T., Yu H., Shyr Y., Patil T., Horn L., McCoach C., Kelly K., Doebele R.C., Camidge D.R. (2018). First-line Chemotherapy Responsiveness and Patterns of Metastatic Spread Identify Clinical Syndromes Present Within Advanced KRAS Mutant Non-Small-cell Lung Cancer with Different Prognostic Significance. Clin. Lung Cancer.

[35] Cui W., Franchini F., Alexander M., Officer A., Wong H.L., IJzerman M., Desai J., Solomon B.J. (2020). Real-world outcomes in KRAS G12C mutation positive non-small cell lung cancer. Lung Cancer.

[36] Gibert J., Clavé S., Hardy-Werbin M., Taus Á., Rocha P., Longarón R., Piquer G., Chaib I., Carcereny E., Morán T. (2020). Concomitant genomic alterations in KRAS mutant advanced lung adenocarcinoma. Lung Cancer.

[37] Spira A.I., Tu H., Aggarwal S., Hsu H., Carrigan G., Wang X., Ngarmchamnanrith G., Chia V., Gray J.E. (2021). A retrospective observational study of the natural history of advanced non-small-cell lung cancer in patients with KRAS p.G12C mutated or wild-type disease. Lung Cancer.

[38] Cai D., Hu C., Li L., Deng S., Yang J., Han-Zhang H., Li M. (2020). The prevalence and prognostic value of KRAS co-mutation subtypes in Chinese advanced non-small cell lung cancer patients. Cancer Med..

[39] Arbour K.C., Rizvi H., Plodkowski A.J., Hellmann M.D., Knezevic A., Heller G., Yu H.A., Ladanyi M., Kris M.G., Arcila M.E. (2021). Treatment Outcomes and Clinical Characteristics of Patients with KRAS-G12C-Mutant Non-Small Cell Lung Cancer. Clin. Cancer Res..

[40] Mazieres J., Drilon A., Lusque A., Mhanna L., Cortot A.B., Mezquita L., Thai A.A., Mascaux C., Couraud S., Veillon R. (2019). Immune checkpoint inhibitors for patients with advanced lung cancer and oncogenic driver alterations: Results from the IMMUNOTARGET registry. Ann. Oncol..

[41] Sebastian M., Eberhardt W.E.E., Hoffknecht P., Metzenmacher M., Wehler T., Kokowski K., Alt J., Schütte W., Büttner R., Heukamp L.C. (2021). KRAS G12C-mutated advanced non-small cell lung cancer: A real-world cohort from the German prospective, observational, nation-wide CRISP Registry (AIO-TRK-0315). Lung Cancer.

[42] Jeanson A., Tomasini P., Souquet-Bressand M., Brandone N., Boucekine M., Grangeon M., Chaleat S., Khobta N., Milia J., Mhanna L. (2019). Efficacy of Immune Checkpoint Inhibitors in KRAS-Mutant Non-Small Cell Lung Cancer (NSCLC). J. Thorac. Oncol..

[43] Hedgeman E., Nørgaard M., Dalvi T., Pedersen L., Hansen H.P., Walker J., Midha A., Shire N., Boothman A.M., Fryzek J.P. (2021). Programmed cell death ligand-1 expression and survival in a cohort of patients with non-small cell lung cancer receiving first-line through third-line therapy in Denmark. Cancer Epidemiol..

[44] Aredo J.V., Padda S.K., Kunder C.A., Han S.S., Neal J.W., Shrager J.B., Wakelee H.A. (2019). Impact of KRAS mutation subtype and concurrent pathogenic mutations on non-small cell lung cancer outcomes. Lung Cancer.

[45] Renaud S., Guerrera F., Seitlinger J., Reeb J., Voegeli A.C., Legrain M., Mennecier B., Santelmo N., Falcoz P.E., Quoix E. (2018). KRAS-specific Amino Acid Substitutions are Associated with Different Responses to Chemotherapy in Advanced Non-small-cell Lung Cancer. Clin. Lung Cancer.

[46] Ricciuti B., Brambilla M., Cortellini A., De Giglio A., Ficorella C., Sidoni A., Bellezza G., Crinò L., Ludovini V., Baglivo S. (2020). Clinical outcomes to pemetrexed-based versus non-pemetrexed-based platinum doublets in patients with KRAS-mutant advanced non-squamous non-small cell lung cancer. Clin. Transl. Oncol..

[47] Park S., Kim J.Y., Lee S.H., Suh B., Keam B., Kim T.M., Kim D.W., Heo D.S. (2017). KRAS G12C mutation as a poor prognostic marker of pemetrexed treatment in non-small cell lung cancer. Korean J. Intern. Med..

[48] Passiglia F., Cappuzzo F., Alabiso O., Bettini A.C., Bidoli P., Chiari R., Defferrari C., Delmonte A., Finocchiaro G., Francini G. (2019). Efficacy of nivolumab in pre-treated non-small-cell lung cancer patients harbouring KRAS mutations. Br. J. Cancer.

[49] Lefebvre C., Martin E., Hendriks L.E.L., Veillon R., Puisset F., Mezquita L., Ferrara R., Sabatier M., Filleron T., Dingemans A.C. (2020). Immune checkpoint inhibitors versus second-line chemotherapy for patients with lung cancer refractory to first-line chemotherapy. Respir. Med. Res..

[50] Karatrasoglou E.A., Chatziandreou I., Sakellariou S., Stamopoulos K., Kavantzas N., Lazaris A.C., Korkolopoulou P., Saetta A.A. (2020). Association between PD-L1 expression and driver gene mutations in non-small cell lung cancer patients: Correlation with clinical data. Virchows Arch..

[51] Liu C., Zheng S., Jin R., Wang X., Wang F., Zang R., Xu H., Lu Z., Huang J., Lei Y. (2020). The superior efficacy of anti-PD-1/PD-L1 immunotherapy in KRAS-mutant non-small cell lung cancer that correlates with an inflammatory phenotype and increased immunogenicity. Cancer Lett..

[52] Kim J.H., Kim H.S., Kim B.J. (2017). Prognostic value of KRAS mutation in advanced non-small-cell lung cancer treated with immune checkpoint inhibitors: A meta-analysis and review. Oncotarget.

[53] Lee C.K., Man J., Lord S., Cooper W., Links M., Gebski V., Herbst R.S., Gralla R.J., Mok T., Yang J.C. (2018). Clinical and Molecular Characteristics Associated with Survival Among Patients Treated with Checkpoint Inhibitors for Advanced Non-Small Cell Lung Carcinoma: A Systematic Review and Meta-analysis. JAMA Oncol..

[54] Peters S., Gettinger S., Johnson M.L., Jänne P.A., Garassino M.C., Christoph D., Toh C.K., Rizvi N.A., Chaft J.E., Carcereny Costa E. (2017). Phase II Trial of Atezolizumab As First-Line or Subsequent Therapy for Patients with Programmed Death-Ligand 1-Selected Advanced Non-Small-Cell Lung Cancer (BIRCH). J. Clin. Oncol..

[55] Alessi J.V., Ricciuti B., Jiménez-Aguilar E., Hong F., Wei Z., Nishino M., Plodkowski A.J., Sawan P., Luo J., Rizvi H. (2020). Outcomes to first-line pembrolizumab in patients with PD-L1-high (≥50%) non-small cell lung cancer and a poor performance status. J. Immunother. Cancer.

[56] Sun L., Hsu M., Cohen R.B., Langer C.J., Mamtani R., Aggarwal C. (2021). Association Between KRAS Variant Status and Outcomes with First-line Immune Checkpoint Inhibitor-Based Therapy in Patients with Advanced Non-Small-Cell Lung Cancer [published correction appears in JAMA Oncol. 2021 Jul 1;7(7):1074]. JAMA Oncol..

[57] Salgia R., Pharaon R., Mambetsariev I., Nam A., Sattler M. (2021). The improbable targeted therapy: KRAS as an emerging target in non-small cell lung cancer (NSCLC). Cell. Rep. Med..

[58] Jänne P.A., van den Heuvel M.M., Barlesi F., Cobo M., Mazieres J., Crinò L., Orlov S., Blackhall F., Wolf J., Garrido P. (2017). Selumetinib Plus Docetaxel Compared with Docetaxel Alone and Progression-Free Survival in Patients with KRAS-Mutant Advanced Non-Small Cell Lung Cancer: The SELECT-1 Randomized Clinical Trial. JAMA.

[59] Goldman J.W., Mazieres J., Barlesi F., Dragnev K.H., Koczywas M., Göskel T., Cortot A.B., Girard N., Wesseler C., Bischoff H. (2020). A Randomized Phase III Study of Abemaciclib Versus Erlotinib in Patients with Stage IV Non-small Cell Lung Cancer with a Detectable KRAS Mutation Who Failed Prior Platinum-Based Therapy: JUNIPER. Front. Oncol..

[60] Pujol J.L., Vansteenkiste J., Paz-Ares Rodríguez L., Gregorc V., Mazieres J., Awad M., Jänne P.A., Chisamore M., Hossain A.M., Chen Y. (2021). Abemaciclib in Combination with Pembrolizumab for Stage IV KRAS-Mutant or Squamous NSCLC: A Phase 1b Study. JTO Clin. Res. Rep..

[61] Bradbury P.A., Morris D.G., Nicholas G., Tu D., Tehfe M., Goffin J.R., Shepherd F.A., Gregg R.W., Rothenstein J., Lee C. (2018). Canadian Cancer Trials Group (CCTG) IND211: A randomized trial of pelareorep (Reolysin) in patients with previously treated advanced or metastatic non-small cell lung cancer receiving standard salvage therapy. Lung Cancer.

[62] Shire N.J., Klein A.B., Golozar A., Collins J.M., Fraeman K.H., Nordstrom B.L., McEwen R., Hembrough T., Rizvi N.A. (2020). STK11 (LKB1) mutations in metastatic NSCLC: Prognostic value in the real world. PLoS ONE.

[63] Papillon-Cavanagh S., Doshi P., Dobrin R., Szustakowski J., Walsh A.M. (2020). STK11 and KEAP1 mutations as prognostic biomarkers in an observational real-world lung adenocarcinoma cohort. ESMO Open.

[64] Nie W., Gan L., Wang X., Gu K., Qian F.F., Hu M.J., Zhang D., Chen S.Q., Lu J., Cao S.H. (2021). Atezolizumab prolongs overall survival over docetaxel in advanced non-small-cell lung cancer patients harboring STK11 or KEAP1 mutation. Oncoimmunology.

[65] Bange E., Marmarelis M.E., Hwang W.T., Yang Y.X., Thompson J.C., Rosenbaum J., Bauml J.M., Ciunci C., Alley E.W., Cohen R.B. (2019). Impact of *KRAS* and *TP53* Co-Mutations on Outcomes After First-Line Systemic Therapy Among Patients with STK11-Mutated Advanced Non-Small-Cell Lung Cancer. JCO Precis. Oncol..

[66] Facchinetti F., Bluthgen M.V., Tergemina-Clain G., Faivre L., Pignon J.P., Planchard D., Remon J., Soria J.C., Lacroix L., Besse B. (2017). LKB1/STK11 mutations in non-small cell lung cancer patients: Descriptive analysis and prognostic value. Lung Cancer.

[67] Arbour K.C., Jordan E., Kim H.R., Dienstag J., Yu H.A., Sanchez-Vega F., Lito P., Berger M., Solit D.B., Hellmann M. (2018). Effects of Co-occurring Genomic Alterations on Outcomes in Patients with KRAS-Mutant Non-Small Cell Lung Cancer. Clin. Cancer Res..

[68] Biton J., Mansuet-Lupo A., Pécuchet N., Alifano M., Ouakrim H., Arrondeau J., Boudou-Rouquette P., Goldwasser F., Leroy K., Goc J. (2018). TP53, STK11, and EGFR Mutations Predict Tumor Immune Profile and the Response to Anti-PD-1 in Lung Adenocarcinoma. Clin. Cancer Res..

[69] Ricciuti B., Arbour K.C., Lin J.J., Vajdi A., Vokes N., Hong L., Zhang J., Tolstorukov M.Y., Li Y.Y., Spurr L.F. (2022). Diminished Efficacy of Programmed Death-(Ligand)1 Inhibition in STK11- and KEAP1-Mutant Lung Adenocarcinoma Is Affected by KRAS Mutation Status. J. Thorac. Oncol..

[70] Gerber D.E., Camidge D.R., Morgensztern D., Cetnar J., Kelly R.J., Ramalingam S.S., Spigel D.R., Jeong W., Scaglioni P.P., Zhang S. (2020). Phase 2 study of the focal adhesion kinase inhibitor defactinib (VS-6063) in previously treated advanced KRAS mutant non-small cell lung cancer. Lung Cancer.

[71] Herbst R., Lopes G., Kowalski D., Kasahara K., Wu Y., Castro G., Cho B., Turna H., Cristescu R., Aurora-Garg D. (2019). Association of KRAS mutational status with response to pembrolizumab monotherapy given as first-line therapy for PDL1-positive advanced non-squamous NSCLC in KEYNOTE-042. Ann. Oncol..

[72] Gadgeel S., Rodriguez-Abreu D., Felip E., Esteban E., Speranza G., Reck M. (2019). KRAS mutational status and efficacy in KEYNOTE-189: Pembrolizumab (pembro) plus chemotherapy (chemo) vs placebo plus chemo as first-line therapy for metastatic non-squamous NSCLC. Ann. Oncol..

[73] Gadgeel S., Rodriguez-Abreu D., Felip E., Esteban E., Speranza G., Reck M., Hui R., Boyer M., Garon E. (2020). Pembrolizumab plus pemetrexed and platinum vs placebo plus pemetrexed and platinum as first-line therapy for metastatic nonsquamous NSCLC: Analysis of KEYNOTE-189 by STK11 and KEAP1 status [abstract]. Proceedings of the Annual Meeting of the American Association for Cancer Research 2020.

[74] Cho B.C. Genetic Mutations and Immunotherapy vs. Chemotherapy in KEYNOTE-042. Proceedings of the AACR Virtual Annual Meeting.

[75] Ferdinandos S., Cecilia A.K., David H.M., Dinkar P.P., Elpi M.M., Mark M.A., Christopher M.J., Jessica H., Justin F.G., Anastasios D. (2019). Association of STK11/LKB1 genomic alterations with lack of benefit from the addition of pembrolizumab to platinum doublet chemotherapy in non-squamous non-small cell lung cancer. J. Clin. Oncol..

[76] Johnson M., De Langen J., Waterhouse D., Mazieres J., Dingemans A.C. Sotorasib versus docetaxel for previously treated non-small cell lung cancer with KRAS G12C mutation: CodeBreaK 200 phase III study. Proceedings of the 2022 Annual Meeting of the European Society for Medical Oncology.

[77] Jänne P.A., Riely G.J., Gadgeel S.M., Heist R.S., Ou S.I., Pacheco J.M., Johnson M.L., Sabari J.K., Leventakos K., Yau E. (2022). Adagrasib in Non-Small-Cell Lung Cancer Harboring a KRASG12C Mutation. N. Engl. J. Med..

